# Derivation of Human Toxicokinetic Parameters and Chemical-Specific Adjustment Factor of Citrinin Through a Human Intervention Trial and Hierarchical Bayesian Population Modeling

**DOI:** 10.3390/toxins17080382

**Published:** 2025-07-31

**Authors:** Lia Visintin, Camilla Martino, Sarah De Saeger, Eugenio Alladio, Marthe De Boevre, Weihsueh A. Chiu

**Affiliations:** 1 Centre of Excellence in Mycotoxicology and Public Health, Faculty of Pharmaceutical Sciences, Ghent University, 9000 Ghent, Belgium; sarah.desaeger@ugent.be (S.D.S.); marthe.deboevre@ugent.be (M.D.B.); 2 Doping Control Laboratory, Faculty of Medicine and Health Sciences, Ghent University, 9000 Ghent, Belgium; 3 Department of Chemistry, University of Torino, 10125 Torino, Italy; camilla.martino@unito.it (C.M.);; 4 Department of Biotechnology and Food Technology, University of Johannesburg, Gauteng 2092, South Africa; 5 Regional Anti-Doping and Toxicological Centre, 10043 Orbassano, Italy; 6 Department of Veterinary Physiology and Pharmacology, Interdisciplinary Faculty of Toxicology, Texas A&M University, College Station, TX 77840, USA

**Keywords:** citrinin, toxicokinetics, chemical-specific adjustment factors

## Abstract

Background: Citrinin (CIT) is a mycotoxin produced by various fungi contaminating stored cereals and fruits. While biomonitoring and food occurrence data indicate widespread exposure, its public health risks remain unclear due to the lack of human toxicokinetic (TK) data. Methods: A UHPLC-MS/MS method was validated for CIT quantification in capillary blood (VAMS Mitra^®^ tips), feces, and urine obtaining LLOQs ≤ 0.05 ng/mL. A human TK study was conducted following a single oral bolus of 200 ng/kg bw CIT. Individual capillary blood (VAMS Mitra^®^ tips), feces, and urine samples were collected for 48 h after exposure. Samples were analyzed to determine CIT’s TK profile. Results: TK modeling was performed using a multi-compartmental structure with a hierarchical Bayesian population approach, allowing robust parameter estimation despite the lack of standards for CIT metabolites. Conclusions: The derived TK parameters align with preliminary human data and significantly advance CIT exposure assessment via biomonitoring. A human inter-individual toxicokinetic variability (HK_AF_) of 1.92 was calculated based on the derived AUC, indicating that EFSA’s current default uncertainty factor for TK variability is adequately protective for at least 95% of the population.

## 1. Introduction

**Figure 1:**
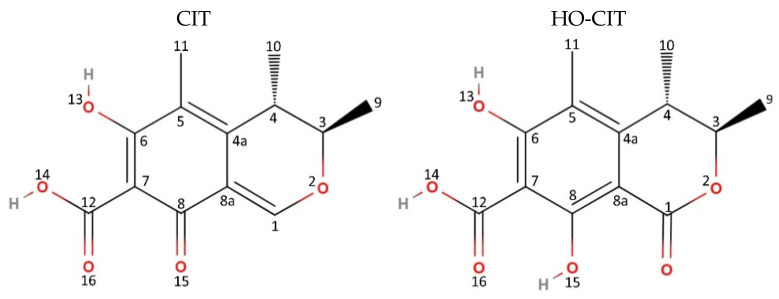
Chemical structure of citrinin (CIT) produced by *Aspergillus*, *Penicillium*, and *Monascus species* and its major human metabolite dihydrocitrinone (HO-CIT). Structure created with MolView.org.

Citrinin (CIT, Figure 1) is a mycotoxin produced by various species of *Aspergillus*, *Penicillium*, and *Monascus* that has emerged as a significant concern for food safety due to its widespread occurrence in human foods, particularly in stored cereals, fruits, and other plant-derived products [1,2,3]. Numerous in vitro studies have revealed a wide spectrum of toxic effects associated with CIT exposure. The primary mechanism underlying CIT’s toxicity is oxidative stress, characterized by an imbalance between the production of reactive oxygen species (ROS) and the cell’s antioxidant defense capacity [4], which leads to various cellular damages, including lipid peroxidation, mitochondrial dysfunction, and apoptosis [5,6,7,8]. Additionally, CIT was demonstrated to impact reproductive cells, reducing cell viability, proliferation, and developmental capacity [9,10]. CIT was found to be highly absorbed by the human intestinal epithelium and to be a permeability glycoprotein nonsubstrate; once absorbed by the gastrointestinal (GI) tract, it cannot be pumped out, hence leading to bioconcentration or biomagnification in the body [11]. On the other hand, the GI tract is expected to be an important excretion pathway, as demonstrated by a study conducted in rats in which up to 16% of CIT was recovered in feces [12,13]. Through in vivo studies, key toxicodynamic parameters were established for CIT, including a no-observed-adverse-effect level (NOAEL) of 20 µg/kg body weight (bw) per day in rats and, accordingly, a derived level of no concern for nephrotoxicity in humans at 0.2 μg/kg bw per day [14]. The kidney is the primary target organ for CIT toxicity, although hepatotoxic effects have also been reported. While animal studies indicated that CIT induces renal adenomas, it is not considered mutagenic [3]. The International Agency for Research on Cancer (IARC) has classified CIT as a Group III compound, meaning that it is not classifiable as to its carcinogenicity to humans [15].

Several human biomonitoring (HBM) studies have reported citrinin exposures both above and below the EFSA reference level. For instance, a study conducted in Bangladesh found exposure levels ranging from 2 to 4663 ng/kg bw [16]. In Germany, another HBM study reported exposures between 2 and 461 ng/kg bw, with the highest levels observed in children [17]. In contrast, two Belgian studies found lower exposure levels, between 0.05 and 7.5 ng/kg bw, once again with highest levels recorded for children [18,19]. Despite the growing evidence regarding CIT’s toxic effects, a significant gap in the understanding of its toxicokinetics in humans remains. Several studies showed that CIT undergoes conversion into dihydrocitrinone (HO-CIT, Figure 1) after *per oral* administration, which is subsequently excreted as the major urinary metabolite [16,20,21,22,23]. A preliminary toxicokinetic study in humans demonstrated urinary excretion of both CIT and HO-CIT, with cumulative excretion ranging from 32.9% to 70.8% within 24 h [16]. Human biomonitoring (HBM) studies recorded urinary concentrations of HO-CIT 3 to 17 times higher than the one of CIT [19,21,24]. However, the biotransformation pathways, inter-individual variability of CIT metabolism, and its main toxicokinetic (TK) parameters are not yet fully known.

The occurrence of CIT in food commodities worldwide has been documented, but the limited availability of comprehensive occurrence data makes it challenging to accurately estimate human exposure and conduct thorough risk assessments [14,18]. Biomonitoring studies have detected trace levels of CIT and HO-CIT in human urine samples across various countries, with prevalence rates ranging from 59 to 94% for CIT and 1.5 to 84% for HO-CIT [19,20,22,24,25,26,27]. Nevertheless, the lack of accurate TK data for CIT makes the assessment of the risk associated to this mycotoxin difficult. Moreover, the discontinued availability of analytical standards of HO-CIT makes it challenging to further characterize the ADME properties of CIT and conduct full risk assessments.

The present study aims to support exposure and risk assessment by determining the TK parameters of CIT in humans and the chemical-specific adjustment factors (CSAF) for human inter-individual toxicokinetic variability (HK_AF_) [28]. To achieve this, sample preparation and ultra-high performance liquid chromatography coupled with tandem mass spectrometry (UHPLC-MS/MS) methods were optimized and validated for the accurate and sensitive quantification of CIT in capillary blood collected via volumetric absorptive microsampling (VAMS), urine, and feces. The methods were then applied to samples obtained from a human TK trial to obtain the human TK profiles of CIT. Finally, the results were used to build a multi-compartmental TK model with Bayesian population structure (PopTK) to estimate the toxicokinetic parameters of CIT. The model implemented a hierarchical population structure, a statistical framework which elucidates the connection between population variability and statistical error on the parameters and data, aiming to provide population-level estimates of parameter distributions [29,30]. This structure is used to derive a quantitative depiction of the variability in the kinetic behavior of the compound across a population modeled from individual-level data [31]. Additionally, the PopTK model proposed uses a Bayesian approach which consists in integrating prior knowledge with experimental data. Thus, each parameter is given a prior distribution based on in vivo pig scaled data which is integrated with the experimental data obtained through the human TK trial.

By providing crucial data on CIT toxicokinetics in humans, this research aims to contribute to more accurate risk assessments and informed policy decisions regarding CIT exposure through food.

## 2. Results and Discussion

### 2.1. Sample Preparation and UHPLC-MS/MS Method

#### 2.1.1. Method Validation

The results of the method validation performed for the quantification of CIT in whole blood collected with VAMS Mitra^®^ tips, urine, and feces are reported in Table 1.

The optimal calibration models were evaluated comparing linear and quadratic models and assessing the model’s weight in case of heteroscedastic distribution [32]. The best calibration model was a heteroscedastic quadratic curve with a weight equal to 1/x^2^ for the blood (R^2^ = 0.9926) and a heteroscedastic linear curve with weight 1/x for urine (R^2^ = 0.9951) and feces (R^2^ = 0.9989). The acceptability of the R^2^ (coefficient of determination) of quantification methods can vary depending on the specific application, the type of analytes being measured, and the requirements set by regulatory guidelines. For many analytical applications, including quantification using UHPLC-MS/MS, an R^2^ value above 0.98 is frequently considered adequate [33,34,35]. The LODs for the methods were acceptable for the application and coherent with the calibration range chosen (0.027 ng/mL in blood, 0.005 ng/mL in urine, and 0.002 ng/g in feces). The accuracy of a method is considered optimal if the variation from the reference value is less than ±15% and acceptable if it is less than ±20% [33,35]. For all calibration points in all matrices, the intra- and inter-day bias was consistently <15%, confirming the high accuracy of the methods. Similar results were obtained for the precision (RSD_r_ and RSD_R_) expressed as CV %. Both parameters were <20% for the three matrices, and all calibration points demonstrated that the precision is adequate.

#### 2.1.2. Matrix Effect

Matrix effect (ME) was assessed in terms of signal suppression/enhancement (SSE), apparent recovery (R_A_), and extraction efficiency (R_E_), reported in Table 1. R_E_ was optimal for CIT in blood (108.5%) and acceptable for feces (76.6%), but low for CIT in urine (57.8%), making the use of IS compensation essential. The investigation of the SSE showed an increased signal for CIT in blood and urine, 113.1% and 175.3%, respectively. While as expected, the signal for CIT was suppressed in feces probably because of the complexity of the matrix. The R_A_ was 122.7% in blood, 129.4% in urine, and only 36.5% in feces, demonstrating once again the need to compensate for the matrix effect using an IS. For these reasons, isotope-labelled CIT (^13^C_13_-CIT) was added as IS to each sample before the extraction. The apparent recovery, achieved by considering the responses, i.e., the ratio of the areas of the analyte and the IS corrected by the concentration of the IS, was between 93.0% and 101.33% for all three matrices. This demonstrated that any loss due to incomplete extraction or increment of signal was proportional systematic errors [36]. Therefore, the addition of ^13^C_13_-CIT as IS accounted for the ME preserving the overall recovery and accuracy.

#### 2.1.3. Stability

The stability was tested under storage conditions coherent with the conditions real samples undergo during the toxicokinetic trial [34,37]. The stability test mimicked 5 days of storage with conditions consistent with a domestic environment and a long-term storage at lower temperature. Stability test results for urine and capillary blood collected via VAMS Mitra^®^ tips are reported in Table 2. The stability was investigated at 0.1 ng/mL and 2.5 ng/mL in capillary blood stored at room temperature (20 °C) for 5 days or 4 °C for 21 days. While in urine, the conditions tested were 4 °C for 5 days and −20 °C for 21 days at 0.01 ng/mL and 10 ng/mL. CIT did not show any sign of degradation, with mean differences ranging −8.55–15.00% considering all the conditions tested and both matrices. CIT’s stability was studied in food and revealed to be influenced by several factors such as temperature, presence of reactive compounds, and fermentation processes [38]. Moreover, it was demonstrated to be affected by solvent composition. The presented results show that CIT is stable in urine and dry capillary blood regardless of the storage temperature. Nevertheless, the storage at lower temperatures obtained overall slightly lower mean differences, if compared to the storage with warmer conditions. Therefore, it is advisable to choose the storage with the lowest temperature (≤−20 °C) available especially in case of long-term storage.

### 2.2. Toxicokinetic Modeling

The toxicokinetic modeling involved the integration of multiple statistical approaches and data types within a multi-compartmental framework. Specifically, the PopTK model included compartments for the GI tract, feces, urine, and central blood compartment, covering both CIT and its metabolite HO-CIT. The model employed a hierarchical population structure, allowing for a clear distinction between uncertainty and intra-/inter-individual variability. Additionally, a Bayesian approach was used to fit the model and estimate the TK parameters of CIT. This approach integrated prior distributions for TK parameters derived from allometric scaling of pig data with experimental data from the human toxicokinetic trial. These data were combined through an iterative process until convergence was achieved, yielding the posterior population TK parameters.

#### 2.2.1. TK Profiles and Deterministic Model

**Figure 2:**
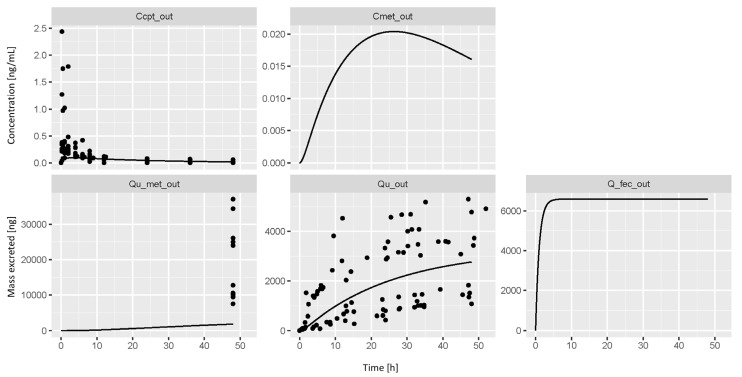
Deterministic model (black line) simulating CIT’s blood concentration (Ccpt_out), CIT’s mass excreted in urine (Qu_out) and feces (Q_fec_out), concentration of HO-CIT in blood (Cmet_out), and mass of HO-CIT excreted in urine (Qu_met_out) represented by the black dots. The simulation was obtained using TK parameters allometrically scaled to humans from pig data [18] in comparison with human data obtained during the toxicokinetic trial. The values for Qu_met_out were estimated based on the total mass of CIT excreted in urine (48 h) and the literature data [16,19,21,24,40].

The analysis of the urine and capillary blood samples obtained from the 10 volunteers revealed that CIT is rapidly absorbed and distributed, but slowly excreted, similarly to pigs [18]. However, at low concentrations, CIT was detectable (>LOD) in urine and capillary blood for up to 48 h with individual maximum concentrations ranging between 0.25–2.44 ng/mL in blood and 1.59–8.00 ng/mL in urine. The cumulative urinary excretion of CIT in 48 h accounted for 22.0 ± 12.3% of the dose administered. The profiles revealed a certain degree of inter-individual variability. Additionally, the cumulative urinary excretion profile showed the typical increment in steps usually associated with enterohepatic circulation, which is coherent with the blood profiles of the volunteers [39]. Previous studies that include the analysis of the fecal samples obtained in rats confirmed the finding according to which the GI tract represents one of the main excretion routes for CIT [12,13]. Specifically, in these studies, the mycotoxin was detected in 11 samples of the 18 collected and could be quantified in 4 of them with concentrations ranging between 0.01–0.631 ng/g of dry feces. CIT was detected in 5 samples with concentrations above the LOD and below the LLOQ (0.01 ng/mL), and in 2 other samples with concentrations higher than the ULOQ (5 ng/g). These profiles are perfectly in line with the preliminary TK data published by Degen et al. (2018) [16], which reported an average excretion of 20.7 ± 17.0% of the dose administered and t_1/2_ in plasma varying between 7.5 h and 13.8 h. The cumulative mass of CIT excreted in urine in 48 h was integrated to the literature data [16,19,21,24,40] reporting HO-CIT/CIT ratios between 3 and 17 to estimate the cumulative mass excreted as HO-CIT by the volunteers in 48 h. The cumulative mass of CIT of each volunteer was multiplied by a random number picked from a normal distribution extending from 3 to 17. The values obtained were truncated to the mass administered and not recovered as CIT in order to maintain the mass balance. The results were used to evaluate a deterministic model built using the toxicokinetic parameters obtained from the allometric scaling of pig data published by Meerpoel et al. (2020) [18]. The results are shown in Figure 2.

The deterministic model obtained is in the correct order of magnitude of the blood profile of CIT, but it does not describe accurately the absorption phase, probably due to the human absorption rate and volume of distribution used, which were estimated from the pig data. In fact, Degen et al. (2018) [16] reported low V_dist_ for CIT in humans, ranging from 0.053 to 0.123 L/kg, while the allometric scaling of pig data resulted in 0.9 L/kg. On the contrary, the urinary excretion profile of CIT is very well predicted, in accordance with a good estimation of the Cl [16]. As expected, the estimated cumulative urinary excretion of HO-CIT was not correctly simulated. This is possibly due to the lack of TK parameters for HO-CIT that were set equal to the ones of CIT and/or to a poor estimation of the fraction excreted as HO-CIT in 48 h, which was based on the literature HBM data.

#### 2.2.2. PopTK Model Fit and Posterior Predictions

The convergence diagnostic (Ȓ) was <1.2 for all individual and population parameters, demonstrating that the model reached convergence. The prior and posterior distributions of the population parameters’ means and standard deviations were compared (Figure S1). The posterior distributions of population means and standard deviations related to CIT parameters were more narrow than the corresponding prior distributions, confirming that the model was informative in updating these prior distributions. The parameters related to HO-CIT reflect the prior distributions due to the unavailability of experimental data to these data (Figure S1). Table 3 contains the 90% CI of population geometric mean (GM) and geometric standard deviation (GSD) of the TK parameters derived from the posterior distribution in comparison with the preliminary values obtained by Degen et al. (2018) [16]. The fraction excreted in urine (k_ufrac_) and the half-life (t_1/2_) were consistent with the ones reported in the literature. On the other hand, values published for clearance and volume of distribution were approximately 4-fold lower than the ones previously obtained. Allometric scaling from pig data represented a good starting point for the derivation of the posterior parameters’ distribution, even though there are clear interspecies differences, e.g., in the volume of distribution values. The HK_AF_ calculated as described in equation (1) resulted in a value of 1.92, which is lower than the default uncertainty factor for human variability in toxicokinetics (=3.16). Therefore, the level of no concern for nephrotoxicity in humans established by EFSA using the default factor is conservative and should sufficiently cover human toxicokinetic variability to at least 95% of the population.

**Figure 3:**
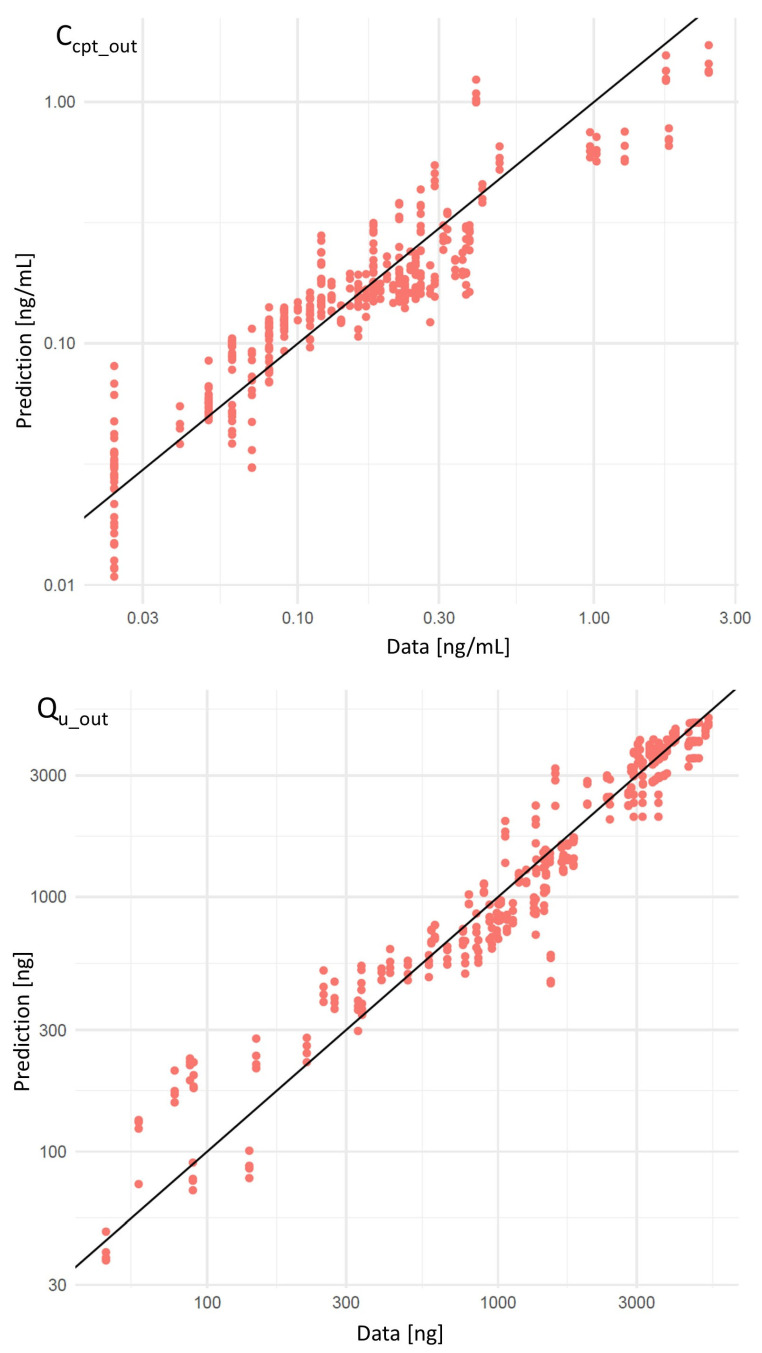
Correlation between predicted values and observed experimental values for the capillary blood concentration of CIT (Ccpt_out) and its mass excreted in urine (Qu_out) across all volunteers.

The model’s fit was evaluated qualitatively through scatter plots between the predicted and experimental data obtained in urine and blood for CIT across all volunteers together (Figure 3) and for each volunteer separately (Figure S2). For both matrices and all volunteers, the correlation was linear, indicating a good fit. The cross-correlation plot of each parameter was also evaluated to disprove the hypothesis of (anti-)correlation between parameters. The plots are reported in Figure S3.

**Figure 4:**
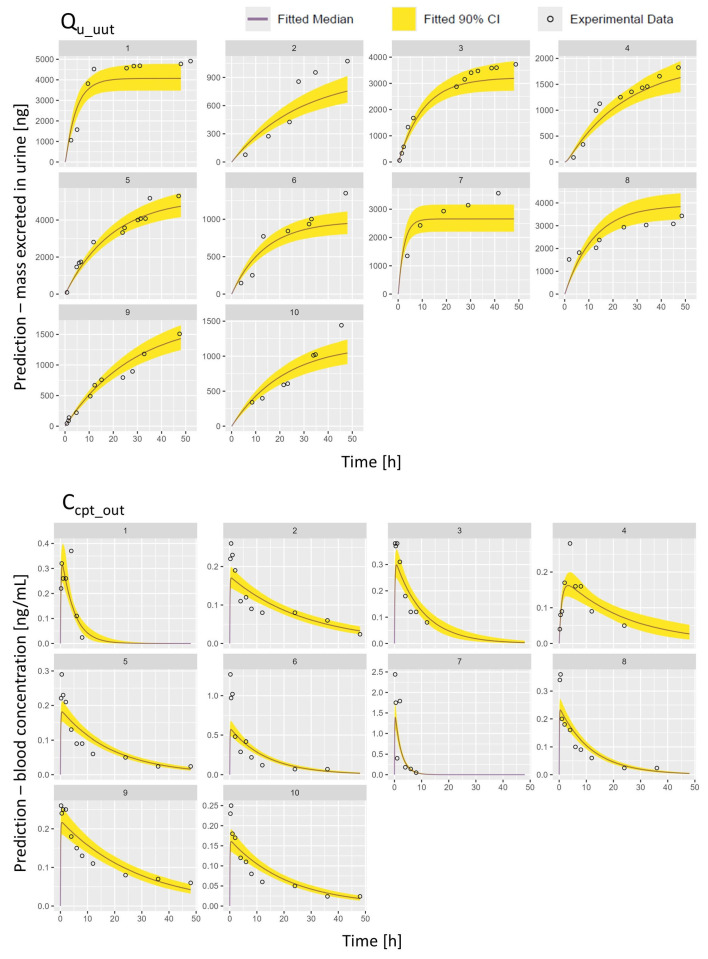
Posterior predictions (median and 90% CI) compared to experimental data concentration of CIT in urine (Q_u_out_) and blood (C_cpt_out_).

The model’s predictive performance was further checked comparing the model’s prediction and the TK profiles obtained experimentally for CIT. Figure 4 shows, for each volunteer, the predictions of the blood concentration and urinary excretion profiles of CIT, along with the 90% confidence interval (CI) displayed as yellow ribbon. Examining the different profiles obtained, it is clear that inter-individual differences both in the rate of clearance and the total excretion of CIT are present. 

## 3. Limitations, Strengths, and Conclusions

A method for the quantification of citrinin (CIT) in capillary blood (VAMS Mitra^®^ tips) and urine was successfully validated. The method reached excellent sensitivity for the quantification of CIT at ultra-trace levels in both matrices (LLOQ_urine_ and LLOQ_feces_ = 0.01 ng/mL, LLOQ_blood_ = 0.05 ng/mL). In addition, the stability of CIT in urine and capillary blood collected using VAMS Mitra^®^ tips was evaluated (Table 2), and matrix effects were assessed (Table 1). The validated method provided a solid foundation for characterizing the toxicokinetic profiles of CIT in blood and urine samples collected during the human trial involving 10 volunteers. The collection of the three biological matrices and their analysis provided essential data for the application of toxicokinetic modeling. The toxicokinetic modeling, calibrated using a hierarchical Bayesian population structure approach, demonstrated a good prediction of CIT levels in blood and urine, offering valuable insights into the compound’s behavior in the human body. The TK parameters estimated for CIT (Table 1) were consistent with the preliminary parameters published by Degen et al. (2018) [16], although values obtained for clearance and volume of distribution were approximately 4-fold higher. Based on the GSD associated with the AUC, the HK_AF_ was calculated, demonstrating that the default uncertainty factor for toxicokinetic human variability used to derive the EFSA health-based guidance value is sufficiently protective of at least 95% of the population [41].

Several limitations were overcome through the use of TK modeling. The major constraints were caused by the absence of a commercially available analytical standard for HO-CIT, which hinders the determination of the toxicokinetic profiles for this metabolite and influences the comprehensiveness of the data used for the model. This gap in analytical capabilities echoes a broader challenge in the field of HBM of mycotoxins, an issue consistently highlighted by the scientific community. Another limitation stemmed from a deficit of the design of the trial. While fecal samples were collected and analyzed, the lack of data on fecal mass excreted restricts our understanding of the complete excretion profile of CIT. While it was confirmed that CIT is excreted in feces [12], this omission left a gap in our ability to calculate the fraction excreted via the GI tract. Despite these constraints, the model fits well with the available CIT data thanks to the application of the Bayesian approach.

The challenges encountered in this study highlight several important considerations for future research on this topic. The critical need for comprehensive data collection and analysis is evident. Future studies should prioritize obtaining a complete set of biological samples, including feces, and developing analytical methods for all relevant metabolites, including HO-CIT. The successful use of allometric scaling from pig data as a starting point for human parameter estimation demonstrates the value of animal studies in toxicokinetic modeling. However, it also emphasizes the need for careful validation and refinement using human data to ensure the model’s accuracy and relevance to human physiology.

The findings of this study contribute significantly in refining risk assessment practices for CIT by providing compound-specific human toxicokinetic (TK) data derived from an intervention trial, rather than relying solely on animal studies and default assumptions. Specifically, the use of a human PopTK model allows for more accurate internal exposure estimations from HBM data, enabling reverse dosimetry approaches that are directly relevant to human exposure conditions. By quantifying inter-individual variability in human TK parameters rather than assuming default uncertainty factor for toxicokinetics (HK_AF_), the study also challenges the broad application of EFSA’s default factor of 10^1/2^ for human inter-individual TK variability. The observed variability in human TK is narrower than these defaults, suggesting that chemical-specific adjustment factors (CSAFs) could replace default HK_AF_, leading to more tailored and potentially less conservative risk characterization. To date, risk assessment of mycotoxins is mainly performed based on external exposure assessment due to the lack of human biomonitoring guidance values (HBGV) and TK parameters. Although the model could still be implemented to refine the parameters associated with HO-CIT, the model closed some important knowledge gaps using a robust and data-driven approach. As new data become available, updating the model will be crucial to enhance its performance in estimating CIT TK parameters and broadening its applicability. On the other hand, missing HBGV values still hinder the use of HBM data and of the results obtained in a regulatory context. Nevertheless, the data provided will aid the transition from external to internal exposure assessment driven by regulatory agencies and the scientific community.

## 4. Materials and Methods

### 4.1. Materials

Five milligrams of CIT solid standard and 12.2 μg of isotope-labelled CIT (^13^C_13_-CIT) were obtained from Fermentek (Jerusalem, Israel). The mycotoxin and isotope-labelled form were dissolved in 5 and 1 mL of acetonitrile (CH_3_CN) to reach the final concentration of 1 mg/mL and 12.2 μg/mL, respectively. From the individual stock solutions kept at −20 °C, two working solutions of CIT with concentrations of 10 ng/mL and 1 μg/mL and two internal standard (IS) solutions of ^13^C_13_-CIT with concentrations of 10 ng/mL and 100 ng/mL were prepared in methanol (CH_3_OH). Ultra-pure water was obtained from an Arium^®^ Pro water system from Sartorius (Brussels, Belgium). CH_3_OH and CH_3_CN LC-MS grade (99.95%) were purchased from BioSolve (Valkenswaard, The Netherlands). Glacial acetic acid (CH_3_COOH, 100%) and formic acid (HCOOH, 98–100%) were supplied by Merck (Darmstadt, Germany), as well as sodium acetate (CH_3_COONa) and ammonium acetate (CH_3_COONH_4_). Sodium chloride (NaCl) and magnesium sulphate (MgSO_4_) were purchased from VWR chemicals (Leuven, Belgium). VAMS devices (Mitra^®^ tips) were obtained from Trajan Scientific and Medical (Torrance, CA, USA). BD Microtainer Contact-activated lancets and urine sample containers were purchased from Novolab (Geraardsbergen, Belgium). Urine samples for method validation were gently donated from 5 healthy volunteers and stored at −20 °C until use. EDTA-anticoagulated blood samples for method validation purposes were supplied by Rode Kruis Vlaanderen (Ghent, Belgium) and kept at −80 °C until use.

### 4.2. Human Intervention Trial

The human intervention study was conducted as described by Visintin & Lu et al. (2025) [42]. The trial was performed in compliance with Belgian and European legislation and the Declaration of Helsinki. The presented protocol was approved by the Ethical Committee of Ghent University Hospital (UZGent, Gent, Belgium) through an amendment to the original dossier B670201630414. Informed consent was obtained from every volunteer prior to participation. Ten volunteers were recruited and instructed to adhere to a specific diet developed to minimize CIT intake during the 5-day trial. Sex, age, body weight, body mass index (BMI), and ethnicity of the volunteers are reported in Table S1. This involved avoiding commonly CIT-contaminated foods such as breakfast cereals, seeds and nuts, pears, pasta, rice, and spices [14]. On the third day, before breakfast, volunteers consumed 5 mL of an aqueous bolus containing CIT at the level of no concern (200 ng/kg bw per day), as determined by EFSA [14]. This dose was selected because it is both representative of real-life human exposure levels, as demonstrated by multiple HBM studies [16,17,18,19], and considered safe. Over the following 48 h, urine, blood, and fecal samples were collected recording for each individual sample the void volume (for urine) and collection time. The blood collection was performed following a sampling schedule at 0, 15, 30 min; and 1, 2, 4, 6, 8, 12, 24, 36, and 48 h. VAMS Mitra^®^ tips were chosen over traditional blood withdrawal for their ability to allow the collection of multiple serial blood samples with minimal burden and invasiveness for the volunteers. In fact, blood sampling using VAMS Mitra^®^ tips was performed autonomously by the participants, eliminating the need for repeated visits to the research facility, multiple venipunctures, or cannula insertion.

In total, 120 blood, 97 urine, and 18 fecal samples were collected and stored at −80 °C to ensure sample stability [43]. The samples were then extracted and analyzed for the quantification of CIT by matrix-matched calibration with internal standard.

### 4.3. Sample Preparation

#### 4.3.1. Whole Blood (VAMS Mitra^®^ Tips)

The calibration curve was built by adding the CIT standard at 1 μg/mL to 1 mL blank EDTA-blood aliquots to achieve final concentrations of 0–0.05–0.1–0.5–1.0–2.5–5 ng/mL. Calibrants were prepared by dipping the VAMS tip into the spiked whole blood and let air-dry in the dark for at least 3 h. The tip was then removed from the plastic support and placed into an Eppendorf tube. ^13^C_13_-CIT standard at 100 ng/mL was added as IS to the dried blood-saturated tips to reach the final concentration of 1 ng/mL. Before the extraction, an additional 30 min were allowed to ensure complete evaporation of the IS solvent. Two-hundred and fifty μL of extraction solvent consisting of CH_3_OH/H_2_O (90/10, *v*/*v*) were added to the samples. The samples were then sonicated for 30 min and shaken for 30 min at room temperature using an overhead shaker (Agilitec, J. Toulemonde, and Cie, Paris, France). The tips were removed from the Eppendorf tube, and the extracts were evaporated until dryness under a gentle N_2_ stream (40 °C), reconstituted in 50 μL of injection solvent (50/50 CH_3_OH/H_2_O *v*/*v*, 1% CH_3_COOH), and centrifuged at 10,000× *g* for 5 min. All samples were then transferred into UHPLC vials and centrifuged a second time.

#### 4.3.2. Urine

The extraction of CIT in urine was performed as described by Ouhibi et al. (2020) [44]. The calibration curve was built by spiking 2 mL of blank urine aliquots with the 10 ng/mL or 1 μg/mL CIT standard to reach final concentrations of 0–0.01–0.05–0.1–0.5–1.0 and 5.0 ng/mL. The samples were spiked with the IS at 100 ng/mL until the final concentration of 1 ng/mL. Finally, a different volume of CH_3_OH was added to each calibrant to obtain the same final CH_3_OH percentage. The samples were extracted using the salting-out assisted liquid–liquid extraction (SALLE). The spiked samples were diluted in 18 mL of extraction solvent (CH_3_CN/H_2_O/HCOOH, 52/45/3, *v*/*v*/*v*), and 4 g of anhydrous magnesium sulphate and 1 g of sodium chloride were added. Next, the samples were vortexed for 1 min and shaken for 30 min at room temperature using an overhead shaker (Agilitec, J. Toulemonde, and Cie, Paris, France). The two phases were separated by centrifugation for 10 min at 4000× *g* (Sigma 3–16PK, Osterode am Harz, Germany), and 7 mL of the organic phase was evaporated until dryness under a gentle N2 stream at 35 °C. Reconstitution of the residues was performed in 250 μL of injection solvent (CH_3_OH/H_2_O/CH_3_COOH, 50/49/1, *v*/*v*/*v*) before transferring the samples into UHPLC vials. Finally, the samples were centrifuged for 10 min at 10,000× *g* (Sigma 3–16PK, Osterode am Harz, Germany).

#### 4.3.3. Feces

The extraction of CIT in feces was performed adapting the method of Puntscher et al. (2019) [45]. The calibration curve was built by spiking 100 mg of freeze-dried blank feces with the 10 ng/mL CIT standard to reach final concentrations of 0–0.01–0.05–0.1–0.5–1.0–2.5 and 5.0 ng/mL. The samples were spiked with the IS at 100 ng/mL until the final concentration of 1 ng/mL. The samples were kept in a cool and dark place for 30 min to let the solvent evaporate. The samples were extracted using 900 µL of extraction solvent (CH_3_CN/CH_3_OH/H_2_O, 4/4/2). Next, the samples were vortexed for 30 sec to homogenize them, sonicated for 15 min in an ice bath, and shaken for 30 min at room temperature using an overhead shaker (Agilitec, J. Toulemonde, and Cie, Paris, France). The solid phase was separated by centrifugation for 15 min at 10,000× *g* (Sigma 3–16PK, Osterode am Harz, Germany); 650 µL of the supernatant was transferred in a new reaction tube and diluted with 50 µL of extraction solvent. The samples were kept at −20 °C for at least 60 min and filtered using centrifugal filters (Durapore^®^ polyvynilidene fluoride 22 µm centrifugal filters (Merk Millipore, Cork, Ireland) for 10 min at 10,000× *g*. Finally, the samples were transferred in UHPLC vials prior to analysis.

### 4.4. UHPLC-MS/MS Analysis and Method Validation

Chromatographic separation was achieved using an Acquity i-Class UHPLC system (Waters^®^, Manchester, UK) equipped with an Acquity HSS T3 100 × 2.1 mm UHPLC column (1.8 μm particle size) and Acquity Vanguard HSS T3 10 × 2.1 mm UHPLC pre-column (1.8 μm particle size), both from Waters^®^ (Manchester, UK). Column and autosampler temperatures were set at 35 °C and 10 °C, respectively. The injection volume was 10 μL. Gradient elution was established with an aqueous mobile phase (mp) consisting of 5% CH_3_OH, 1% CH_3_COOH, and 5mM of CH_3_COONH_4_ in ultra-pure H_2_O (mp A) and an organic mp consisting of 2% H_2_O, 1% CH_3_COOH, and 5 mM of CH_3_COONH_4_ in CH_3_OH (mp B) at a flow rate of 0.4 mL/min. The gradient program started at 2% mp B that was increased to 8% in 4 min. At 4.10 min, the % mp B was brought to 65% and subsequently to 100% at 8 min. This condition was kept for 3 min until the polarity of the mp was switched to 2% mp B at 11.10 min. Finally, the column was let to equilibrate for 1 min, for a total run time of 12 min. Analytes were detected using a Waters Xevo^®^ TQ-XS tandem quadrupole mass spectrometer (Waters, Manchester, United Kingdom) equipped with an ESI interface and operating in Multiple Reaction Monitoring (MRM) mode. The desolvation gas temperature was 600 °C and the flow was 1000 L/h. The cone curtain gas and collision flow were 150 L/h and 7.0 bar. The source temperature was kept at 130 °C. N_2_ was used as nebulizer and curtain gas, while the collision gas was Ar. The optimized capillary voltage was 0.80 kV for ESI^-^ mode while the MRM parameters were optimized specifically for CIT and ^13^C_13_-CIT to maximize the sensitivity of the method. The optimized MRM parameters and the retention times (Rt) of the analytes are reported in Table 4 for each compound. Masslynx^®^ and Targetlynx^®^ software version 4.2 (Waters Corp., Milford, CT, USA) were used for data acquisition and processing.

The methods were validated following the guidelines regarding mycotoxin analysis of the European Commission (EC) Decision No. 2021/808, Commission Implementing Regulation (EU) No. 2023/2782, ISO 5725-1:2023, SANTE/12089/2016 identification criteria, and ICH M10 for bioanalytical method validation [33,34,46,47]. The data processing was performed implementing the pipeline published by Desharnais et al. (2017) [32]. The validation protocol foresees the repetition of three calibration curves in three different days, providing a total amount of nine replicates. Additional experiments were performed to determine the matrix effect and stability of the analytes in the matrices considered. The data were used to evaluate the heteroscedasticity, different weights, and linear/quadratic calibration curve models. Moreover, goodness-of-fit of the linear regression (coefficient of determination, R^2^), intra- and inter-day accuracy (bias %) and precision (RSD_r_ and RSD_R_, CV %), limit of detection (LOD), signal suppression enhancement (SSE), apparent recovery (R_A_), extraction efficiency (R_E_), and calibration range (LLOQ-ULOQ) were determined. Finally, specificity, selectivity, ion abundance repeatability, retention time consistency, and carry-over were evaluated. The LOD was calculated considering the confidence interval of the intercept derived from the nine calibration curves, i.e., the signal of a blank sample. The goodness-of-fit of the regression was assessed by calculating the coefficient of determination (R^2^) [48] between the spiked concentrations of the analytes and the corresponding instrument response, which was calculated as the ratio of the analyte peak area to the IS peak area, multiplied by the concentration of the IS. Specificity and selectivity were assessed by evaluating the presence of interfering chromatographic peaks with S/N > 3 in representative blank samples. Identification criteria for the analyte were the presence of all qualifying ions and their relative abundance (±30%) at the expected retention time (±0.05 min). The carry-over was evaluated analyzing pure injection solvent after the highest calibration point into the LC-MS/MS system. The matrix effect (ME) was measured in terms of signal suppression enhancement (SSE), apparent recovery (R_A_), and extraction efficiency (R_E_) that were assessed following the approach described by Sulyok et al. (2006) [36]. The stability of the analyte during the human toxicokinetic trial and analytical procedure is a prerequisite to obtain reliable TK profiles for modeling [34,37]. The stability was investigated at two concentration levels in duplicate to simulate high- and low-concentration scenarios in urine and capillary blood collected via VAMS Mitra^®^ tips. The urine was spiked and stored at 4 °C for 5 days and −20 °C for 21 days, while the VAMS devices were stored at 20 °C for 5 days and 4 °C for 21 days. The hypothesis of influence of hematocrit (Hct) was previously tested, demonstrating that the recovery from VAMS Mitra^®^ tips is not hematocrit dependent [49].

### 4.5. PopTK Modeling

**Figure 5:**
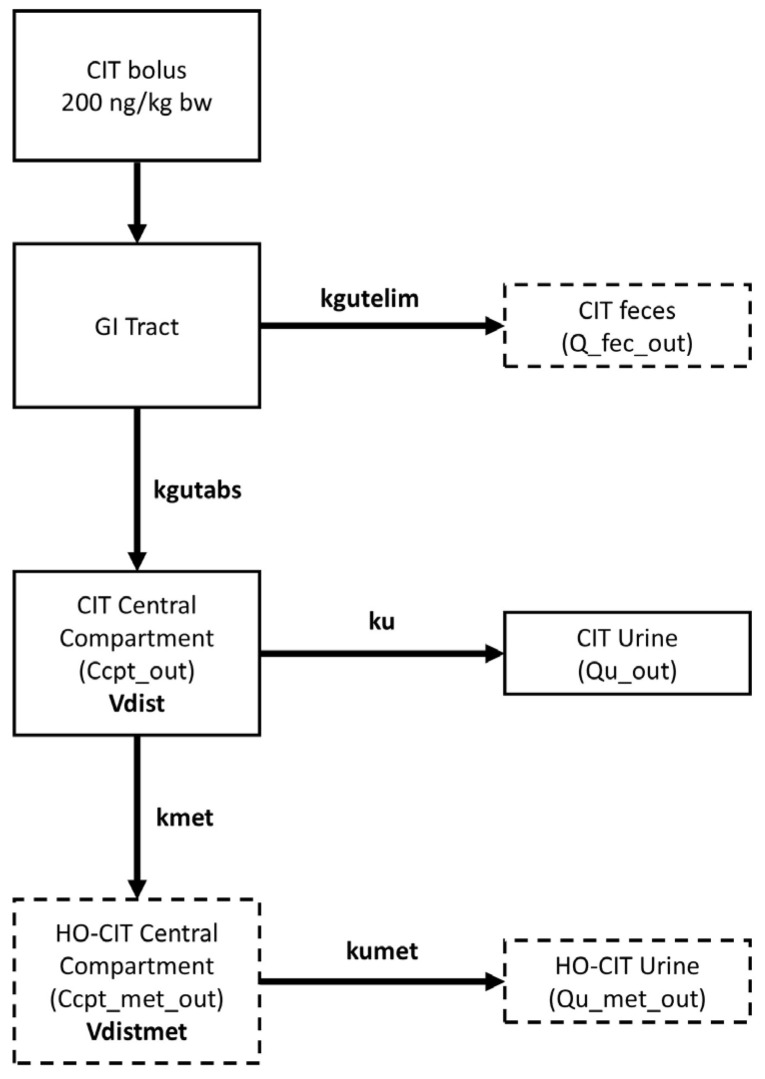
Structure of the multi-compartmental toxicokinetic model used for CIT. The abbreviations reported represent the gastrointestinal tract (GI tract), volume of distribution of CIT (V_dist_), volume of distribution of HO-CIT (V_distmet_), elimination rate constant via GI tract (k_gutelim_), absorption rate constant (k_gutabs_), urinary excretion rate constant (k_u_), metabolic rate constant (k_met_), urinary excretion rate constant of HO-CIT (k_umet_), mass of CIT excreted in feces (Q__fec_out_), concentration of CIT in the central compartment (C_cpt_out_), concentration of HO-CIT in the central compartment (C_cpt_met_out_), mass of CIT excreted in urine (Q_u_out_), and mass of HO-CIT excreted in urine (Q_u_met_out_). The dashed compartments represent matrices for which experimental data are currently unavailable.

The multi-compartmental structure of the PopTK model reported in Figure 5 was chosen based on the literature and experimental data available. The model consists of a GI tract and fecal, urinary, and central compartments for CIT and HO-CIT. From the GI tract, CIT is eliminated with rate k_gutelim_ and absorbed to systemic circulation, or central compartment, with rate k_gutabs_. From the central compartment, CIT is excreted in urine with rate k_u_, or metabolized with rate k_met_. The metabolite HO-CIT is eliminated from its own central compartment into urine with rate k_umet_. The ratio between the amount in the body and the concentration in blood is given by the volume of distribution V_dist_ and V_distmet_, for CIT and HO-CIT, respectively. All elimination rates are expressed in h^−1^ and volume of distribution in L/kg. All parameters were natural log-transformed for fitting. Due to the absence of analytical standards for HO-CIT, C_cpt_met_out_ could not be measured in blood samples experimentally and Q_u_met_out_ was estimated based on Q_u_out_ and the literature data. Due to a limitation of the trial’s design, the mass eliminated via the GI tract could not be calculated since the total fecal mass was not recorded by the volunteers.

#### 4.5.1. Hierarchical Bayesian Population Model

A hierarchical Bayesian population model was used for TK model-fitting and to determine model parameters, uncertainty, and inter-individual variability as previously described by Lu et al. (2023) and Visintin & Lu et al. (2025) [42,50,51]. The prior distributions of the model parameters were retrieved by allometric scaling from data obtained through a pig toxicokinetic trial published by Meerpoel et al. (2020) [52]. The mean value of the parameters reported for female and male pigs was used. Unfortunately, since the values of the gut elimination rate (k_gut_), fraction of CIT eliminated in urine (k_ufrac_), and elimination rate of HO-CIT in urine (k_umet_) were not reported in the literature, a default value of 0.5 was set for these parameters. For the same reason, the priors for the volume of distribution (V_distmet_) and clearance of HO-CIT (Cl_totmet_) were set equal to the volume of distribution (V_dist_) and total clearance of CIT (Cl_tot_), respectively. The parameter setting of prior distributions is detailed in Table 5. The TK model-fitting was carried out performing MCMC simulation using GNU MCSim v6.1.0 software [53] in the R environment [54]. The posterior distributions were derived integrating in the model the experimental data obtained from the human intervention trial performed for CIT. All input and generated files are included in the GitHub repository (https://github.com/liavisintin/TK_CIT). Four independent MCMC chains were run with 11k iterations each. Subsequently, the convergence of the chains was assessed by visual evaluation of the posterior parameter distributions and evaluation of $\hat{R}$ (≤1.2) [55].

#### 4.5.2. Model Fit and Predictions for Human Toxicokinetics

The population and individual TK predictions were evaluated using the comparison with the experimental data obtained from the human intervention trial. Scatter plots between predicted and observed experimental values were used to check qualitatively the accuracy of model calibration. Posterior distributions of the population parameters were plotted to evaluate their plausibility given the prior distributions. The posterior parameter distributions at both population and individual levels were used to generate model predictions of the blood concentration and urinary excretion profiles of CIT. The individual administered CIT dose, body weight, mass of CIT excreted in urine, and the concentration of CIT in capillary blood at each collection time point were defined for each subject to guide the modeling of the profiles in time. At the population level, parameter values were sampled from the population parameters to reflect uncertainty in their means and variances.

### 4.6. Adjustment Factor for Human Variability in Toxicokinetics (HK_AF_)

In the context of hazard characterization, animal toxicity data are used to define the health-based guidance values or toxicological reference points for a xenobiotic by applying a default uncertainty factor of 100 which accounts for interspecies and inter-individual differences in toxicodynamics and toxicokinetics [56]. Under the assumption that effects resulting from sub-chronic or chronic exposure would normally be related to the AUC, the CSAF for human variability in TK (HK_AF_) can be determined for a compound on the basis of the human variability associated with the AUC. The most suitable toxicokinetic parameter for the definition of CSAF is the AUC for a dose interval at steady state, which is replaced by the AUC after administration of a single dose extrapolated to infinity in typical cases in which the analyte does not induce or inhibit its own metabolism [31]. Accordingly, to replace the default factor of 10^½^ = 3.16, HK_AF_ associated with the 95th percentile of sensitivity was calculated as shown in the following Equation (1):

(1) $${HK}_{AF}={{GSD}_{AUC}}^{Q_{norm}\left(0.95\right)}$$

where GSD_AUC_ is the geometric standard deviation of AUC for the population and Q_norm_(0.95) is the z-score associated with a 95% probability.

## Figures and Tables

**Table 1:** Results of the method validation for the quantification of citrinin (CIT) in urine, capillary blood collected via VAMS Mitra^®^ tips, and feces. The performances of the method are reported as determination coefficient (R^2^), limit of detection (LOD, ng/mL or ng/g), calibration range expressed as interval Lower Limit of Quantification (LLOQ, ng/mL or ng/g)-Upper Limit of Quantification (ULOQ, ng/mL or ng/g), signal suppression enhancement (SSE, %), apparent recovery (R_A_, %), extraction efficiency (R_E_, %), intra- and inter-day bias (%), repeatability (RSD_r_, %), and intermediate precision (RSD_R_, %). The results were obtained by testing 3 calibration curves for 3 days (*n* = 9). IS = internal standard.

Matrix	R^2^	LOD	Calibration Range	SSE	R_A_		R_e_	Intra-Day Bias		Inter-Day Bias		RSD_r_		RSD_R_	
		ng/mL *	LLOQ-ULOQ	%	Without IS	With IS	%	LLOQ	ULOQ	LLOQ	ULOQ	LLOQ	ULOQ	LLOQ	ULOQ
			ng/mL *		%	%		%	%	%	%	%	%	%	%
Urine	0.9951	0.005	0.01–10	175.4	129.4	101.3	57.8	7.5	1.8	9.1	1.7	11.3	4.1	15.4	4.5
Blood	0.9926	0.027	0.05–2.5	113.1	122.7	94.7	108.5	−9.4	−0.1	−9.3	0.22	14.6	9.1	15.1	16.9
Feces	0.9989	0.002	0.01–5	47.7	36.5	93.0	76.6	−0.1	0.3	1.1	0.3	5.7	2.5	5.9	2.4

* The concentrations for feces are expressed in ng/g of freeze-dried sample.

**Table 2:** Results of the stability test of citrinin (CIT) in urine and capillary blood collected via VAMS Mitra^®^ tips. Results were obtained with different storing condition at two concentration levels analyzed in duplicate. The stability is reported in terms of mean difference between the nominal and the measured concentration after storage quantified using a freshly prepared matrix-matched calibration and internal standard.

Mean Difference Urine (%)				Mean Difference Capillary Blood (%)			
0.01 ng/mL	10 ng/mL	0.01 ng/mL	10 ng/mL	0.1 ng/mL	2.5 ng/mL	0.1 ng/mL	2.5 ng/mL
−20 °C 21 Days	−20 °C 21 Days	4 °C 5 Days	4 °C 5 Days	4 °C 21 Days	4 °C 21 Days	20 °C 5 Days	20 °C 5 Days
15.00	0.10	−1.73	0.03	6.87	14.12	4.84	−8.55

**Table 3:** Post-distribution values obtained for population models and TK parameters of citrinin (CIT). t_1/2_ = half-life, k_el_ = total elimination rate, t_max_ = time to reach C_max_, C_max_ = maximum blood concentration, AUC = area under the curve, Cl_tot_ = clearance CIT, Cl_met_ = clearance HO-CIT, V_dist_ = volume of distribution CIT, V_distmet_ = volume of distribution HO-CIT, F_gutabs_ = fraction absorbed by the gastrointestinal tract, k_gutelim_ = elimination rate via the gastrointestinal tract, k_ufrac_ = fraction of CIT excreted in urine.

Parameter	Unit	Preliminary TK Parameters (Degen et al., 2018) [16]	Population Posterior Distributions Median [90% CI]	
			GM	GSD
t_1/2_	h	7.5–13.8	9.33 [6.43–13.53]	1.29 [1.20–1.52]
k_el_	h^−1^	n.a.	0.074 [0.051–0.108]	1.29 [1.20–1.52]
T_max_ *	h	n.a.	0.63 [0.35–1.15]	1.92 [1.60–2.93]
C_max_ *	ng/mL	n.a.	0.28 [0.15–0.52]	1.99 [1.64–3.09]
AUC **	ng/(L·kg bw)	n.a.	3654.2 [2290.8–5829.1]	1.49 [1.34–1.94]
Cl_tot_	L/(h·kg bw)	0.005–0.007	0.025 [0.020–0.030]	1.51 [1.32–1.71]
Cl_met_	L/(h·kg bw)	n.a.	0.033 [0.005–0.239]	1.19 [0.98–1.45]
V_dist_	L/kg bw	0.052–0.123	0.330 [0.254–0.428]	1.18 [0.95–1.47]
V_distmet_	L/kg bw	n.a.	0.836 [0.136–5.079]	1.72 [1.53–1.95]
F_gutabs_	**-**	n.a.	0.246 [0.093–0.651]	1.17 [0.97–1.41]
k_gutelim_	h^−1^	n.a.	4.141 [2.565–6.686]	1.96 [1.61–2.40]
k_ufrac_	**-**	0.076–0.456	0.351 [0.273–0.451]	1.69 [1.49–1.92]

n.a. = not available, GM = geometric mean, GSD = geometric standard deviation, CI = confidence interval. * Calculated for a dose of 1500 ng/kg bw. ** Calculated from the 10 subjects in the intervention trial.

**Table 4:** Multiple Reaction Monitoring (MRM) parameters used for the quantification of citrinin (CIT) and its internal standard. Retention time (Rt), cone voltage, mass-to-charge ratio of the methanol adduct ions ([M+CH_3_OH-H]^−^ *m*/*z*), mass-to-charge ratio of the product ions *m*/*z*, and collision energy (CE). The letter Q is identifying the quantifier ions.

Analyte	Rt	Cone	[M+CH_3_OH-H]^−^ *m*/*z*	CE	Product Ion *m*/*z*
	(min)	(V)		(eV)	
**CIT**	7.30	30	281.30	25	249.20 (Q)
				15	205.15 *
**^13^** **C_13_-CIT**	7.30	30	294.30	25	262.20 (Q)
				15	217.20

* Quantifier ion for CIT in feces.

**Table 5:** Human toxicokinetic model parameters, natural logarithm values, and prior distribution set based on allometric scaling of pig data obtained by Meerpoel et al. (2020) [41].

Parameter	Description (Unit)	Central Value	Prior Distribution for Population Geometric Mean (Natural Logarithm)
Cl_tot_	Total clearance of CIT (L/Kg·h)	0.04	LogNormal (−3.33, 1.15)
Cl_met_	Clearance of HO-CIT (L/Kg·h)	0.04	LogNormal (−3.33, 1.15)
V_dist_	Volume of distribution of CIT (L/kg)	0.90	LogNormal (−0.10, 1.15)
V_distmet_	Volume of distribution of HO-CIT (L/kg)	0.90	LogNormal (−0.10, 1.15)
k_ufrac_	Fraction of CIT eliminated in urine	0.50	TruncLogNormal (−0.7, 1.15, −4.61, −0.01)
k_umet_	Elimination rate of HO-CIT in urine (h^−1^)	0.50	LogNormal (−0.7, 1.15)
k_tot_	Total elimination rate of CIT (h^−1^)	0.02	LogNormal (−3.96, 1.15)
k_gutelim_	Gut elimination rate (h^−1^)	0.50	LogNormal (−0.7, 1.15)
F_gutabs_	Fraction absorbed	0.17	TruncLogNormal (−1.76, 1.15, −2.3, 0)

Note: LogNormal (geometric mean, geometric standard deviation); TruncLogNormal (geometric mean, geometric standard deviation, minimum, maximum).

## Data Availability

The raw data supporting the conclusions of this article are openly available in https://github.com/liavisintin/TK_CIT. Further inquiries can be directed to the corresponding author.

## Supplementary Materials

The following supporting information can be downloaded at: https://www.mdpi.com/article/10.3390/toxins17080382/s1. Figure S1: Prior and posterior distributions of the population means and standard deviations of model’s parameters with toxicokinetic relevance. Mean (M), standard deviation (SD), elimination rate of CIT via the GI tract (k_gutelim_), fraction of CIT excreted in urine (k_ufrac_), total clearance of CIT (Cl_tot_), clearance of OH-CIT (Cl_met_), volume of distribution of CIT (V_dist_), volume of distribution of OH-CIT (V_distmet_), fraction absorbed via the GI (F_gutabs_). Figure S2: Correlation between predicted values and observed experimental values for the capillary blood concentration of citrinin (Ccpt_out) and its mass excreted in urine (Qu_out) for each volunteer individually. Figure S3: Cross-correlation plot between model’s parameters with toxicokinetic relevance. Fraction absorbed via the GI tract (F_gutabs_), elimination rate of CIT via the GI tract (k_gutelim_), fraction of CIT excreted in urine (k_ufrac_), total clearance of CIT (Cl_tot_), clearance of OH-CIT (Cl_met_), volume of distribution of CIT (V_dist_), volume of distribution of OH-CIT (V_distmet_). Table S1: Sex, age, body weight (bw), body mass index (BMI), and ethnicity of the ten volunteers recruited for participation in the human toxicokinetic trail performed for the investigation of citrinin (CIT).

## Abbreviations

The following abbreviations are used in this manuscript:

^13^C_13_-CIT	Isotopically labelled citrinin
ADME	Absorption, distribution, metabolization, excretion
AUC	Area under the blood concentration-time curve
bw	Body weight
Ccpt_out	Citrinin concentration in the central compartment (blood flow)
Ccpt_met_out	Metabolites concentration in the central compartment (blood flow)
CE	Collision energy
CI	Confidence interval
CIT	Citrinin
Cl_tot_	Clearance of citrinin
Cl_met_	Clearance of citrinin’s metabolites
C_max_	Maximum blood concentration
CSAF	Chemical-specific adjustment factors
CV	Coefficient of variation
EFSA	European Food Safety Authority
ESI	Electrospray ionization
EU	European Union
F_gutabs_	Fraction absorbed via the gastrointestinal tract
GI	Gastrointestinal
GM	Geometric mean
GSD	Geometric standard deviation
HBM	Human biomonitoring
HBGV	Human biomonitoring guidance values
HO-CIT	Dihydrocitrinone
HK_AF_	Human inter-individual toxicokinetic variability
IARC	International Agency for Research on Cancer
ICF	Informed consent form
ICH	International Council for Harmonisation
IS	Internal standard
k_el_	Total elimination rate of citrinin
k_met_	Metabolization rate
k_gutelim_	Gut elimination rate
k_gutabs_	Gut absorption rate
k_u_	Urinary excretion rate
k_ufrac_	Fraction excreted in urinec
k_umet_	Urinary excretion rate of the metabolite
LLOQ	Lower Limit of Quantification
LOD	Limit of Detection
MCMC	Markov chain Monte Carlo
ME	Matrix effect
mp	Mobile phase
MS	Mass spectrometry/spectrometer
MRM	Multiple reaction monitoring
m/z	Mass-to-charge ratio
NOAEL	No-observed-adverse-effect level
PopTK	Population toxicokinetics
Q	Quadrupole
Q_fec_out	Amount of citrinin excreted via the GI tract
Qu_met_out	Amount of citrinin’s metabolites excreted in urine
Qu_out	Amount of citrinin excreted in urine
Ȓ	R-hat convergence diagnostic
R_A_	Apparent recovery
R_E_	Extraction efficiency
ROS	Reactive oxygen species
RSD_R_	Intermediate precision
RSD_r_	Repeatability
Rt	Retention time
SALLE	Salting-out assisted liquid–liquid Extraction
SSE	Signal suppression enhancement
t_1/2_	Half-life
TK	Toxicokinetic
t_max_	Time to reach C_max_
ULOQ	Upper Limit of Quantification
UHPLC	Ultra-high performance liquid chromatography
VAMS	Volumetric absorptive microsampling
V_dist_	Volume of distribution of citrinin
V_distmet_	Volume of distribution of citrinin’s metabolites
