# Supplementary material for: Derivation of Human Toxicokinetic Parameters and Chemical-Specific Adjustment Factor of Citrinin Through a Human Intervention Trial and Hierarchical Bayesian Population Modeling
Source: Toxins (Basel). 2025 Jul 31;17(8):382. doi: 10.3390/toxins17080382 (PMC12390278; doi:10.3390/toxins17080382)
Supplement: Supplementary file 1 [file toxins-17-00382-s001.zip › toxins-3658796-supplementary.pdf]

SUPPLEMENTARY MATERIAL

Figure S1: Prior and posterior distributions of the population means and standard deviations of model's parameters with toxicokinetic relevance. Mean (M), standard deviation (SD), elimination rate of CIT via the GI tract ($k_{gutelim}$), fraction of CIT excreted in urine (k_{ufrac}), total clearance of CIT (Cl_{tot}), clearance of OH-CIT (Cl_{met}), volume of distribution of CIT (V_{dist}), volume of distribution of OH-CIT ($V_{distmet}$), fraction absorbed via the GI (F_{gutabs}).

Figure S2: Correlation between predicted values and observed experimental values for the capillary blood concentration of citrinin (Ccpt_out) and its mass excreted in urine (Qu_out) for each volunteer individually.

Figure S3: Cross-correlation plot between model's parameters with toxicokinetic relevance. Fraction absorbed via the GI tract (F_{gutabs}), elimination rate of CIT via the GI tract ($k_{gutelim}$), fraction of CIT excreted in urine (k_{ufrac}), total clearance of CIT (Cl_{tot}), clearance of OH-CIT (Cl_{met}), volume of distribution of CIT (Vdist), volume of distribution of OH-CIT ($V_{distmet}$).

Table S1: Sex, age, body weight (bw), body mass index (BMI), and ethnicity of the ten volunteers recruited for participation in the human toxicokinetic trail performed for the investigation of citrinin (CIT).

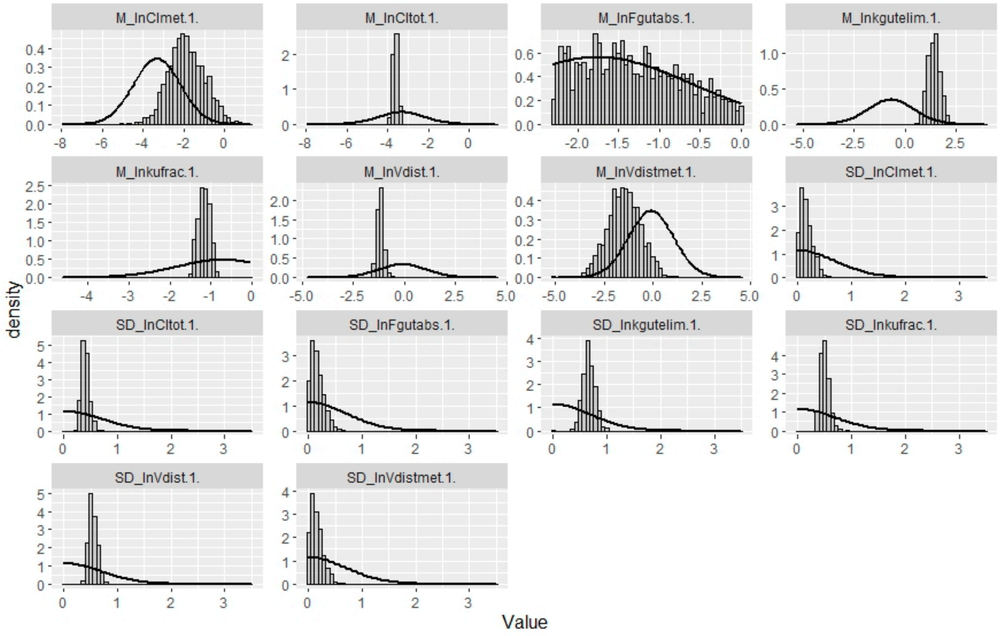

Figure S1: Prior and posterior distributions of the population means and standard deviations of model's parameters with toxicokinetic relevance. Mean (M), standard deviation (SD), elimination rate of CIT via the GI tract ($k_{gutelim}$), fraction of CIT excreted in urine (k_{ufrac}), total clearance of CIT (Cl_{tot}), clearance of OH-CIT (Cl_{met}), volume of distribution of CIT (V_{dist}), volume of distribution of OH-CIT ($V_{distmet}$), fraction absorbed via the GI (F_{gutabs}).

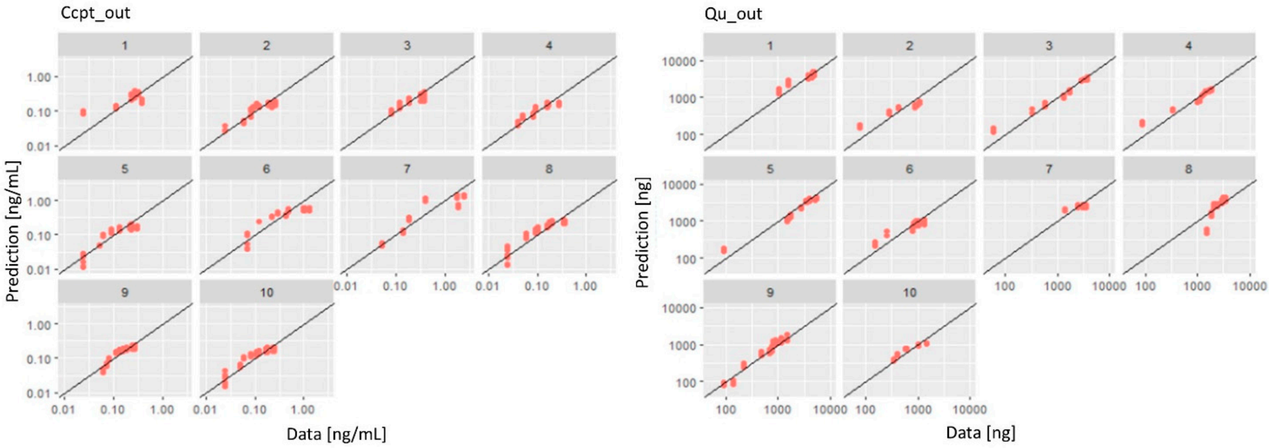

Figure S2: Correlation between predicted values and observed experimental values for the capillary blood concentration of citrinin (Ccpt_out) and its mass excreted in urine (Qu_out) for each volunteer individually.

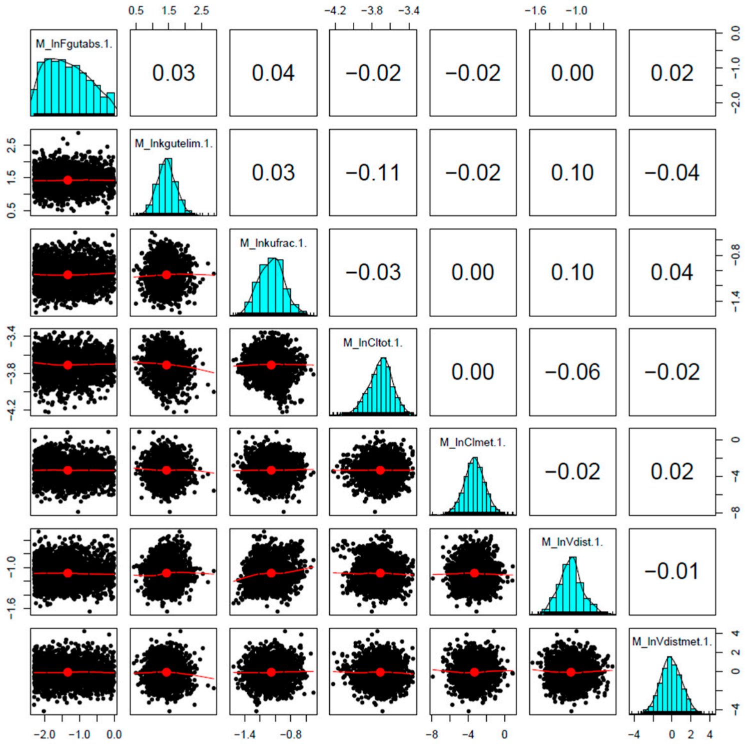

Figure S3: Cross-correlation plot between model's parameters with toxicokinetic relevance. Fraction absorbed via the GI tract (F_{gutabs}), elimination rate of CIT via the GI tract ($k_{gutelim}$), fraction of CIT excreted in urine (k_{ufrac}), total clearance of CIT (Cl_{tot}), clearance of OH-CIT (Cl_{met}), volume of distribution of CIT (Vdist), volume of distribution of OH-CIT ($V_{distmet}$).

Table S1: Sex, age, body weight (bw), body mass index (BMI), and ethnicity of the ten volunteers recruited for participation in the human toxicokinetic trail performed for the investigation of citrinin (CIT).

	Sex	Age (Years)	bw (kg)	BMI (kg/m^2)	Ethnicity
**1**	M	28	85.0	24.8	Caucasian
**2**	F	34	66.0	25.1	Hispanic
**3**	M	22	67.2	21.4	Caucasian
**4**	F	23	65.0	26.7	Caucasian
**5**	M	22	58.0	22.4	Caucasian
**6**	F	22	57.2	21.3	Caucasian
**7**	F	34	68.0	29.8	African
**8**	F	23	52.2	20.6	Caucasian
**9**	F	27	45.0	18.0	Caucasian
**10**	M	32	95.0	25.5	Hispanic
**Avg.**	60% F	26.7	65.8	23.6	-
